# Maladaptive appearance perfectionism and psychosocial distress among cosmetic dermatology patients

**DOI:** 10.3389/fpubh.2026.1829951

**Published:** 2026-06-01

**Authors:** Li-Na Duo, Ji Cheng, Xiao Sun, Ya Sun, Hao-Yue Deng

**Affiliations:** Department of Dermatology & Medical Aesthetics, Chengdu Seventh People’s Hospital (Affiliated Cancer Hospital of Chengdu Medical College), Chengdu, China

**Keywords:** appearance perfectionism, appearance satisfaction, appearance self-esteem, appearance stress, body image concern, cosmetic dermatology, cross-sectional survey, social appearance anxiety

## Abstract

**Background:**

Cosmetic dermatology is expanding rapidly, yet the psychological mechanisms underlying distress in treatment-seeking patients remain insufficiently characterized. This study examines the associations of maladaptive and adaptive appearance perfectionism with key psychological outcomes and tests whether appearance stress functions as an indirect pathway linking maladaptive perfectionism with adverse psychosocial status.

**Methods:**

A single-center hospital-based cross-sectional survey was conducted in the Dermatology Department of Chengdu Seventh People’s Hospital. Adult patients seeking elective cosmetic dermatology care were recruited consecutively before consultation or treatment. Data were collected through a Wenjuanxing electronic questionnaire using established Chinese-language psychometric scales assessing appearance perfectionism, appearance stress, appearance self-esteem, appearance satisfaction, social appearance anxiety, and body image concern. Multiple linear regression models were used to evaluate adjusted associations. Bias-corrected bootstrapping with 5,000 resamples was used to estimate indirect effects.

**Results:**

Of 346 patients approached, 328 were included in the final analytic sample after data cleaning. Mean age was 30.01 ± 6.99 years, and 80.2% were female. Maladaptive appearance perfectionism was significantly associated with lower appearance self-esteem (*B* = −0.483, *β =* −0.516, *p <* 0.001), lower appearance satisfaction (*B* = −0.338, *β =* −0.396, *p <* 0.001), higher social appearance anxiety (*B* = 0.460, *β =* 0.530, *p <* 0.001), and higher body image concern (*B* = 0.468, *β =* 0.542, *p <* 0.001). Adaptive appearance perfectionism was positively associated with appearance self-esteem (*B* = 0.147, *p =* 0.002) and appearance satisfaction (*B* = 0.230, *p <* 0.001), but not with social appearance anxiety or body image concern. Maladaptive appearance perfectionism was strongly associated with appearance stress (*r =* 0.638, *p <* 0.001). Significant indirect effects through appearance stress were observed for appearance self-esteem, appearance satisfaction, social appearance anxiety, and body image concern.

**Conclusion:**

Maladaptive appearance perfectionism is a prominent psychological correlate of distress among cosmetic dermatology patients, and appearance stress appears to be an important indirect pathway through which such perfectionism relates to poorer psychosocial outcomes. Screening for maladaptive appearance perfectionism and appearance-related stress may have clinical value in cosmetic dermatology settings.

## 1 Introduction

The global demand for aesthetic enhancement has surged dramatically, transforming cosmetic dermatology from a niche specialty into a mainstream component of healthcare consumption. This rapid growth is fueled by technological advancements in minimally invasive procedures, increasing social acceptance, and the pervasive influence of digital and social media (1, 2). The cosmetic procedure market continues to expand, with both surgical and non-surgical interventions witnessing rising popularity across diverse demographics and geographies (1, 3). In regions like Saudi Arabia, studies report that nearly a quarter of participants have undergone a cosmetic dermatological procedure, with higher engagement among younger, educated individuals (1). Similarly, among Lebanese women, a significant proportion reported experience with procedures ranging from laser hair removal to rhinoplasty (4). This trend reflects a broader societal shift where investment in appearance is increasingly framed as an investment in self-care and psychological well-being, driving a multi-billion dollar industry focused on aesthetic medicine (5, 6).

Individuals seeking cosmetic dermatological treatments represent a distinct population with specific psychological characteristics and vulnerabilities. Research consistently indicates that this group exhibits heightened concerns regarding body image and physical appearance compared to the general population (7, 8). A significant mental health consideration in this context is body dysmorphic disorder (BDD), a psychiatric condition characterized by a preoccupation with a perceived or slight defect in appearance that causes significant distress and functional impairment (9, 10). The prevalence of BDD is markedly elevated among those seeking cosmetic interventions, with meta-analytic data estimating rates around 19.2% in individuals requesting cosmetic surgery and even higher figures reported in cosmetic dermatology settings (10, 11). Screening positivity for BDD can be particularly high in specific subgroups, such as rhinoplasty candidates (12). This high prevalence underscores the necessity for clinicians to recognize that appearance concerns driving consultation requests may sometimes be pathological in nature, rooted in a distorted self-perception rather than an objective physical flaw (13, 14). Beyond BDD, cosmetic patients often experience elevated levels of general psychological distress, social appearance anxiety, and reduced self-esteem, forming a complex psychological profile that can significantly impact treatment satisfaction and outcomes (7, 15, 16).

Within this psychological landscape, the construct of perfectionism, particularly when directed towards physical appearance, emerges as a critical personality factor. Appearance perfectionism involves setting excessively high standards for one’s looks and engaging in harsh self-evaluation against those standards (17). Contemporary models distinguish between its maladaptive and adaptive dimensions. Maladaptive perfectionism is characterized by a chronic fear of making mistakes, intense self-criticism over perceived imperfections, and doubts about one’s actions, often leading to significant distress (18, 19). In contrast, adaptive perfectionism may involve setting high personal standards without the associated negative self-evaluation and distress when those standards are not fully met. The Physical Appearance Perfectionism Scale (PAPS) operationalizes this construct, often identifying factors like “Worry about Imperfection” reflecting the maladaptive core and “Hope for Perfection” potentially relating to a more adaptive drive (17). This distinction is crucial, as maladaptive appearance perfectionism is robustly linked to body image dissatisfaction, eating disorder symptomatology, and compulsive exercise, whereas adaptive dimensions might correlate with healthier striving behaviors (17, 18). In cosmetic populations, the relentless pursuit of a flawless appearance, driven by maladaptive perfectionism, may fuel repeated procedures and dissatisfaction, as the internal benchmark for “perfection” remains unattainable (19, 20).

The psychological distress generated by the gap between one’s perceived appearance and internalized perfectionistic standards can be conceptualized as appearance-related stress. This construct represents the cognitive and affective burden, including worry, rumination, and negative emotional states, specifically tied to evaluations of one’s physical self (21). Appearance stress is theorized to function as a key indirect pathway, linking dispositional traits like maladaptive perfectionism with negative psychological outcomes. When individuals high in maladaptive perfectionism engage in upward social comparisons, such as viewing idealized images on social media, they are prone to activate negative cognitive coping strategies like rumination and catastrophizing (21). This process amplifies appearance stress, which in turn erodes self-esteem and exacerbates body image concerns. This aligns with general psychological models where perfectionism fosters disordered behaviors through a focused self-concept, with the associated stress and erroneous beliefs about correcting perceived flaws acting as maintaining factors (22, 23). In the context of cosmetic dermatology, appearance stress may manifest as persistent preoccupation with a skin or facial feature, excessive mirror-checking, and significant anxiety in social situations, thereby serving as a critical mediator between a perfectionistic personality style and clinical levels of distress or body image disturbance.

Current literature supports connections between perfectionism, body image, and psychological well-being in appearance-concerned groups. For instance, individuals with high dysmorphic concerns exhibit elevated levels of overall perfectionism, including concern over mistakes and doubts about actions (19). Social media use, particularly engagement with image-based platforms and appearance-focused motivations, is positively associated with body dysmorphic symptoms, a relationship potentially amplified by self-oriented perfectionism (20). Furthermore, low self-esteem is a well-established hallmark of BDD, even after accounting for depressive symptoms (9, 24). Cosmetic surgery has been shown to yield a small-to-moderate improvement in self-esteem post-operatively, particularly following procedures like breast augmentation, though this effect is not universal and is often absent or negative in patients with BDD (5, 16, 25). However, critical gaps persist in the literature specific to dermatology. Most research on appearance perfectionism has been conducted in Western populations or focused on eating disorders, muscle dysmorphia, or cosmetic surgery patients, with limited investigation within the dermatology-specific setting where non-surgical, minimally invasive procedures dominate (17, 26). Additionally, while correlates like social appearance anxiety and self-esteem are often studied separately, their interrelationships with both maladaptive and adaptive facets of appearance perfectionism in a single integrated model are poorly understood within a dermatology patient cohort (27, 28). The potential mediating role of appearance stress in linking perfectionism to outcomes like self-esteem and body image concern has also not been empirically tested in patients actively seeking cosmetic dermatological care, leaving the psychological mechanisms unclear.

The scientific rationale for the present study is therefore multi-faceted. Despite the rapid growth of cosmetic dermatology and the recognized psychological complexity of its patient population, a nuanced understanding of how specific personality dispositions like appearance perfectionism translate into psychological distress remains underdeveloped. Public health relevance stems from the need to identify modifiable psychological risk factors that could inform targeted screening and supportive interventions within dermatology practices, potentially mitigating patient dissatisfaction and poor psychosocial outcomes (29, 30). Clinically, elucidating the pathways is significant for dermatologists and cosmetic practitioners. A clearer grasp of whether appearance stress acts as a critical mediator can guide more effective patient communication, pre-consultation screening, and the appropriate referral of patients who might benefit from psychological support prior to or alongside cosmetic treatment (29, 31). Consequently, this single-center, hospital-based cross-sectional survey aimed to examine the associations of maladaptive and adaptive appearance perfectionism with key psychological outcomes, including appearance self-esteem, appearance satisfaction, social appearance anxiety, and body image concern, among adult patients seeking elective cosmetic dermatology care. Furthermore, it sought to empirically test the hypothesis that appearance stress functions as a significant indirect pathway linking maladaptive appearance perfectionism with the adverse psychological outcomes. By employing validated Chinese-language psychometric scales within a real-world clinical setting in China, this study addresses identified evidence gaps and contributes to a more comprehensive psychological profile of the contemporary cosmetic dermatology patient.

## 2 Materials and methods

### 2.1 Study design and setting

This hospital-based cross-sectional survey study was conducted in the Dermatology Department of Chengdu Seventh People’s Hospital. The department provided outpatient cosmetic dermatology consultation and treatment services for a broad range of appearance-related concerns. Data collection was carried out during the study period from March 1, 2025 to September 1, 2025. Eligible participants were recruited consecutively during routine outpatient visits before physician consultation or treatment.

### 2.2 Participants

Adults presenting for elective cosmetic dermatology care during the study period were screened for eligibility. Participants were included if they were aged 18 years or older, were able to read and understand Chinese, and completed electronic informed consent before survey entry. Participants were excluded if they were unable to complete the questionnaire independently because of severe cognitive impairment, serious communication difficulty, or an acute medical or psychiatric condition that, in the judgment of the attending clinician or study investigator, made participation inappropriate. Consecutive sampling was used throughout the recruitment period to reduce selection bias.

### 2.3 Survey instrument and pilot testing

Data were collected using an electronic survey administered through the Wenjuanxing platform. The survey consisted of two parts: a brief investigator-designed section covering sociodemographic and clinical characteristics, and a set of Chinese-language psychometric instruments selected to assess appearance perfectionism, appearance stress, appearance self-esteem, appearance satisfaction, social appearance anxiety, and body image concern. The psychometric scales were established instruments rather than newly created measures.

Before formal recruitment, the full electronic survey was pilot tested in 50 cosmetic dermatology patients from the same department to evaluate clarity, acceptability, completion time, item order, and technical feasibility of smartphone-based administration. Feedback from pilot participants and research staff was reviewed by the study team, and minor adjustments were made to wording, layout, and response flow to improve readability and usability while preserving the original content and scoring structure of the standardized scales. The pilot phase supported the feasibility of formal implementation in the outpatient clinic.

### 2.4 Data collection procedure

Potentially eligible patients were approached by trained investigators in the clinic waiting area during the recruitment period. After receiving a brief explanation of the study, participants accessed the Wenjuanxing survey by scanning a study-specific QR code with their mobile phone. The first page contained the participant information sheet and electronic informed consent form. Only participants who provided consent were allowed to continue to the questionnaire.

Participants completed the survey anonymously on site before clinical consultation or treatment. An investigator remained available throughout the process to answer procedural questions, clarify item wording when requested, and assist with technical issues, but did not guide or influence participant responses. To improve data quality, the electronic questionnaire was configured to minimize missing responses. Completed questionnaires were reviewed according to prespecified data-cleaning rules. Responses were excluded if they showed clear evidence of invalid completion, including duplicate submissions, logically inconsistent demographic information, or completion times below the minimum threshold established during pilot testing.

### 2.5 Measures

#### 2.5.1 Sociodemographic and clinical variables

The investigator-designed section collected information on age, sex, marital status, educational level, employment status, and monthly income. Clinical variables included prior cosmetic treatment history, primary reason for the current visit, and intended cosmetic dermatology procedure category.

#### 2.5.2 Appearance perfectionism

Appearance perfectionism was assessed using the Physical Appearance Perfectionism Scale (PAPS), a multidimensional Chinese-language scale (53).

#### 2.5.3 Appearance stress

Appearance stress was assessed using the Appearance Stress Scale (ASS), a Chinese-language self-report scale (54).

#### 2.5.4 Psychological outcomes

Psychological outcomes included appearance self-esteem, appearance satisfaction, social appearance anxiety, and body image concern. Appearance self-esteem was assessed using the Appearance Self-Esteem Scale (ASES) and referred to an individual (55). Appearance satisfaction was assessed using the Physical Satisfaction Scale (PSS) and reflected subjective satisfaction (56). Social appearance anxiety was assessed using the Social Appearance Anxiety Scale (SAAS; Chinese version) and represented anxiety (57). Body image concern was assessed using the Body Image Concern Inventory (BICI; Chinese version: Wang et al. 58) and reflected preoccupation.

### 2.6 Sample size estimation

The sample size was estimated *a priori* on the basis of the primary analytic objective, namely testing whether appearance stress showed a statistically significant indirect effect in the association between appearance perfectionism and psychological outcomes within a multivariable regression framework. Using G*Power version 3.1.9.7, a two-sided alpha of 0.05, statistical power of 0.90, and a conservative small-to-moderate effect size of f^2^ = 0.06, the minimum required sample size for linear multiple regression with the main predictor, mediator, and up to eight covariates was 244 participants. Because mediation analyses require stable parameter estimation and because some loss was expected after exclusion of invalid or incomplete questionnaires, the calculated sample size was inflated by approximately 20%. Accordingly, the target sample size was set at no fewer than 300 completed questionnaires, which was considered sufficient to support adjusted association analyses and bootstrap estimation of indirect effects with adequate precision.

### 2.7 Statistical analysis

All statistical analyses were performed using IBM SPSS Statistics for Windows, version 27.0 (IBM Corp., Armonk, NY, USA). Mediation analyses were conducted using the PROCESS macro for SPSS, version 4.2. Descriptive statistics were used to summarize participant characteristics and study variables. Continuous variables were presented as means and standard deviations or medians and interquartile ranges, as appropriate, while categorical variables were presented as frequencies and percentages. Distributional characteristics were assessed by graphical inspection and standard tests of normality.

Internal consistency reliability for each psychometric scale was assessed using Cronbach’s alpha. Bivariate associations among appearance perfectionism, appearance stress, and psychological outcomes were examined using Pearson or Spearman correlation coefficients, as appropriate. Group differences in continuous variables were examined using the independent-samples t test, one-way analysis of variance, Mann–Whitney U test, or Kruskal–Wallis test according to variable distribution and number of comparison groups. Associations between categorical variables were examined using the chi-square test.

To address the first study aim, multiple linear regression models were constructed to examine the associations of maladaptive and adaptive appearance perfectionism with each psychological outcome after adjustment for prespecified covariates, including age, sex, educational level, marital status, prior cosmetic treatment history, and intended procedure category. To address the second study aim, appearance stress was examined as a mediator of the association between maladaptive appearance perfectionism and each psychological outcome. Indirect effects were estimated using bias-corrected bootstrapping with 5,000 resamples. Statistical evidence for an indirect effect was considered present when the 95% bootstrap confidence interval did not include zero. All tests were two-sided, and a *p* value of less than 0.05 was considered statistically significant.

### 2.8 Ethical approval

The study protocol was reviewed and approved by the Ethics Committee of Chengdu Seventh People’s Hospital (approval number 2025KY0009). All participants provided electronic informed consent before completing the survey. Participation was voluntary, and personally identifying information was not retained in the final analytical dataset.

## 3 Results

### 3.1 Participant flow and sample characteristics

**Figure 1:**
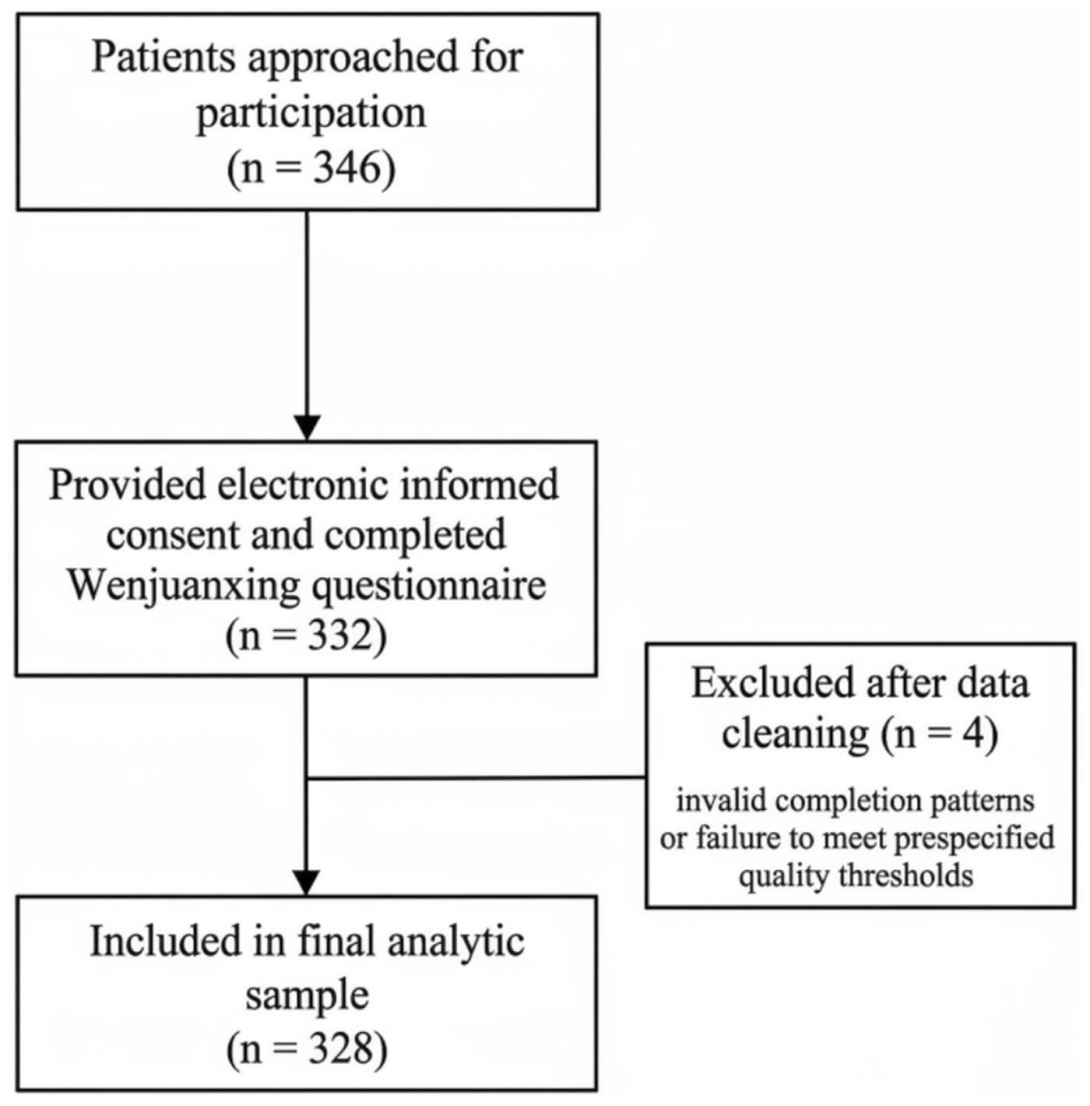
Flow diagram of participant recruitment and inclusion. Flow of participants through screening, survey completion, data cleaning, and final inclusion in the analytic sample. A total of 346 patients were approached, 332 completed the Wenjuanxing questionnaire and provided electronic informed consent, 4 questionnaires were excluded after data cleaning, and 328 participants were retained for the final analysis.

During the study period, 346 patients were approached for participation. Of them, 332 provided electronic informed consent and completed the Wenjuanxing questionnaire. Four questionnaires were excluded during data cleaning because of invalid completion patterns or failure to meet prespecified quality thresholds, leaving 328 participants in the final analytic sample (Figure 1).

The mean age of the final sample was 30.01 years (SD 6.99). Most participants were female (80.2%), single (63.1%), and had a bachelor’s degree (49.7%). Almost half had undergone prior cosmetic treatment (47.6%). The most common presenting reasons for attendance were aging or rejuvenation concerns (25.9%) and facial contour or feature concerns (25.9%), while pigment treatment (19.8%) and acne scar treatment (18.3%) were the most common intended procedure categories (Table 1).

**Table 1:** Sociodemographic and clinical characteristics of the study sample (*N* = 328).

Characteristic	*n* (%) or mean ± SD
Age, years	30.01 ± 6.99
Sex	
Female	263 (80.2)
Male	65 (19.8)
Marital status	
Single	207 (63.1)
Married/other	121 (36.9)
Education	
Junior college or below	95 (29.0)
Bachelor	163 (49.7)
Master or above	70 (21.3)
Employment	
Student	45 (13.7)
Employed	212 (64.6)
Self-employed	38 (11.6)
Unemployed/other	33 (10.1)
Monthly income	
<5,000 RMB	66 (20.1)
5,000 to 9,999 RMB	120 (36.6)
10,000 to 19,999 RMB	100 (30.5)
≥20,000 RMB	42 (12.8)
Prior cosmetic treatment	
No	172 (52.4)
Yes	156 (47.6)
Primary reason for visit	
Acne/scar concerns	76 (23.2)
Pigmentation/skin tone	60 (18.3)
Aging/rejuvenation	85 (25.9)
Facial contour/features	85 (25.9)
Other	22 (6.7)
Intended procedure category	
Laser/energy-based	58 (17.7)
Injectables	52 (15.9)
Acne scar treatment	60 (18.3)
Pigment treatment	65 (19.8)
Skin rejuvenation	57 (17.4)
Combined/other	36 (11.0)

### 3.2 Descriptive statistics and internal consistency of study measures

All psychometric instruments demonstrated acceptable to excellent internal consistency in the analytic sample. Cronbach’s alpha coefficients ranged from 0.785 for adaptive appearance perfectionism to 0.924 for body image concern. Mean scale scores indicated moderate levels of maladaptive appearance perfectionism, appearance stress, social appearance anxiety, and body image concern, alongside mid-range levels of appearance self-esteem and appearance satisfaction (Table 2).

**Table 2:** Descriptive statistics and internal consistency of study measures (*N* = 328).

Measure	No. of items	Cronbach’s α	Total score, mean ± SD	Mean item score, mean ± SD	Observed total score range
Maladaptive appearance perfectionism	6	0.802	19.51 ± 4.30	3.25 ± 0.72	7 to 30
Adaptive appearance perfectionism	6	0.785	21.04 ± 3.83	3.51 ± 0.64	11 to 30
Appearance stress	14	0.881	42.39 ± 8.56	3.03 ± 0.61	18 to 66
Appearance self-esteem	7	0.798	21.32 ± 4.69	3.05 ± 0.67	8 to 32
Appearance satisfaction	11	0.837	33.57 ± 6.72	3.05 ± 0.61	17 to 51
Social appearance anxiety	16	0.908	47.22 ± 9.96	2.95 ± 0.62	24 to 72
Body image concern	19	0.924	58.22 ± 11.77	3.06 ± 0.62	25 to 92

### 3.3 Bivariate associations among appearance perfectionism, appearance stress, and psychological outcomes

**Figure 2:**
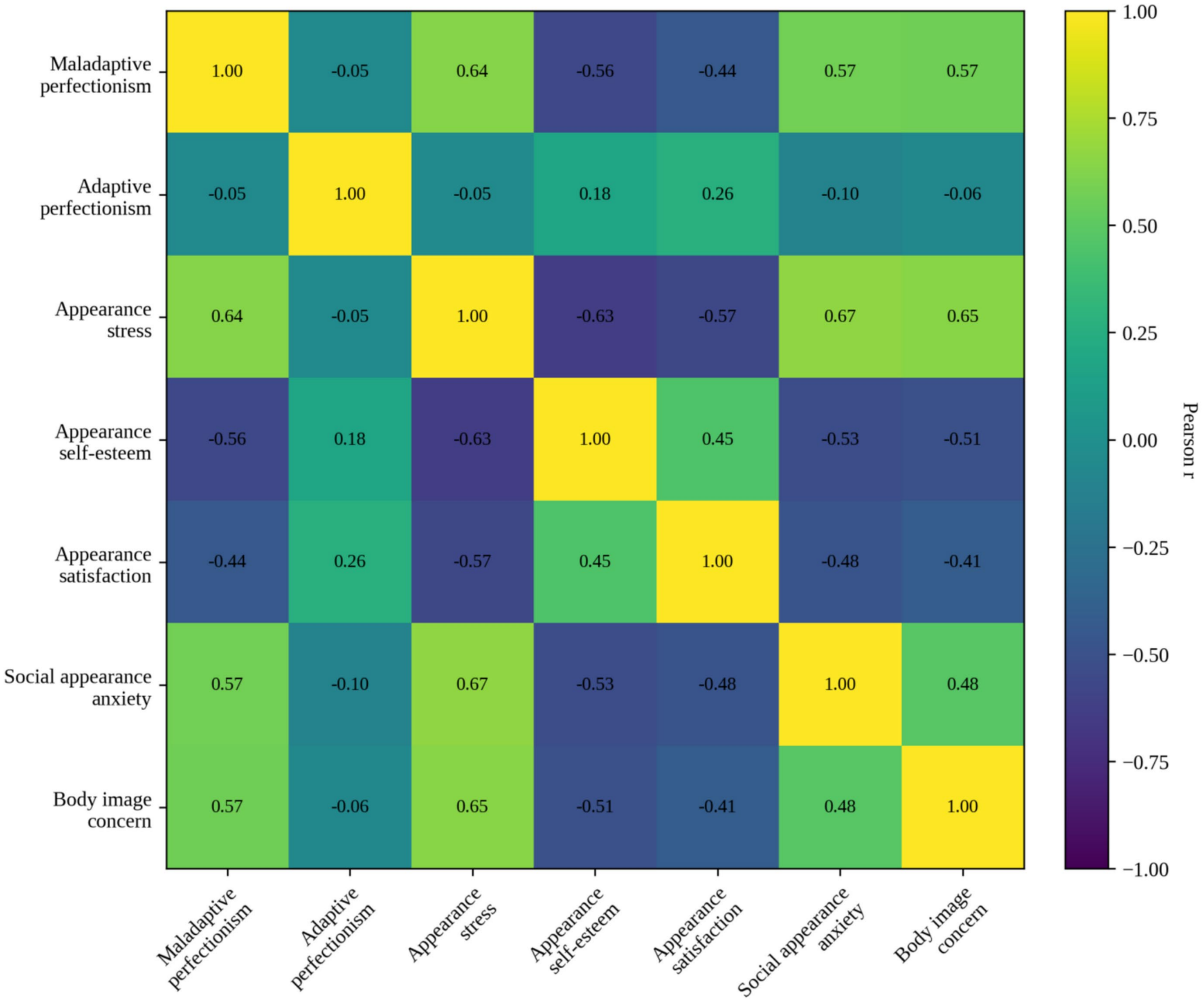
Correlation heatmap of appearance perfectionism, appearance stress, and psychological outcomes: Heatmap of Pearson correlation coefficients among maladaptive appearance perfectionism, adaptive appearance perfectionism, appearance stress, appearance self-esteem, appearance satisfaction, social appearance anxiety, and body image concern in the final analytic sample (*N* = 328). Maladaptive appearance perfectionism was strongly correlated with appearance stress (*r* = 0.638, *p* < 0.001), social appearance anxiety (*r* = 0.572, *p* < 0.001), and body image concern (*r* = 0.566, *p* < 0.001), and negatively correlated with appearance self-esteem (*r* = −0.555, *p* < 0.001) and appearance satisfaction (*r* = −0.441, *p* < 0.001). Appearance stress showed the strongest positive correlations with social appearance anxiety (*r* = 0.667, *p* < 0.001) and body image concern (*r* = 0.647, *p* < 0.001), and strong negative correlations with appearance self-esteem (*r* = −0.632, *p* < 0.001) and appearance satisfaction (*r* = −0.568, *p* < 0.001).

Maladaptive appearance perfectionism showed a strong positive correlation with appearance stress (*r =* 0.638, *p <* 0.001), social appearance anxiety (*r =* 0.572, *p <* 0.001), and body image concern (*r =* 0.566, *p <* 0.001), and a moderate negative correlation with appearance self-esteem (*r =* −0.555, *p <* 0.001) and appearance satisfaction (*r =* −0.441, *p <* 0.001). Adaptive appearance perfectionism was weakly positively associated with appearance self-esteem (*r =* 0.178, *p =* 0.001) and appearance satisfaction (*r =* 0.259, *p <* 0.001), while its correlations with appearance stress, social appearance anxiety, and body image concern were small and not statistically significant. Appearance stress was strongly associated with poorer psychological status across all outcome domains. A correlation heatmap would be an efficient visual summary of the correlation structure at this point (Figure 2), while the full coefficients are presented in Table 3.

**Table 3:** Pearson correlations among key study variables.

Variable	1	2	3	4	5	6	7
1. Maladaptive appearance perfectionism	1						
2. Adaptive appearance perfectionism	−0.052	1					
3. Appearance stress	0.638***	−0.046	1				
4. Appearance self-esteem	−0.555***	0.178**	−0.632***	1			
5. Appearance satisfaction	−0.441***	0.259***	−0.567***	0.447***	1		
6. Social appearance anxiety	0.572***	−0.103	0.667***	−0.530***	−0.480***	1	
7. Body image concern	0.566***	−0.062	0.647***	−0.505***	−0.409***	0.477***	1

**p < 0.01, ***p < 0.001.

### 3.4 Multivariable associations of appearance perfectionism with psychological outcomes

After adjustment for age, sex, educational level, marital status, prior cosmetic treatment history, and intended procedure category, maladaptive appearance perfectionism remained a significant independent correlate of all four psychological outcomes. Higher maladaptive appearance perfectionism was associated with lower appearance self-esteem (*B* = −0.483, SE = 0.043, *β =* −0.516, *p <* 0.001) and lower appearance satisfaction (*B* = −0.338, SE = 0.041, *β =* −0.396, *p <* 0.001), as well as higher social appearance anxiety (*B* = 0.460, SE = 0.039, *β =* 0.530, *p <* 0.001) and higher body image concern (*B* = 0.468, SE = 0.040, *β =* 0.542, *p <* 0.001). By contrast, adaptive appearance perfectionism showed a more selective pattern. It was positively associated with appearance self-esteem (*B* = 0.147, SE = 0.048, *β =* 0.140, *p =* 0.002) and appearance satisfaction (*B* = 0.230, SE = 0.046, *β =* 0.240, *p <* 0.001), but was not significantly associated with social appearance anxiety or body image concern. Model-level explained variance ranged from 30.0% to 38.7% (Table 4).

**Table 4:** Adjusted multiple linear regression models for psychological outcomes.

Outcome	Predictor	B	SE	Standardized *β*	*p* value	Model R^2^
Appearance self-esteem	Maladaptive appearance perfectionism	−0.483	0.043	−0.516	<0.001	0.376
	Adaptive appearance perfectionism	0.147	0.048	0.14	0.002	
Appearance satisfaction	Maladaptive appearance perfectionism	−0.338	0.041	−0.396	<0.001	0.3
	Adaptive appearance perfectionism	0.23	0.046	0.24	<0.001	
Social appearance anxiety	Maladaptive appearance perfectionism	0.46	0.039	0.53	<0.001	0.387
	Adaptive appearance perfectionism	−0.066	0.044	−0.067	0.137	
Body image concern	Maladaptive appearance perfectionism	0.468	0.04	0.542	<0.001	0.355
	Adaptive appearance perfectionism	−0.024	0.045	−0.025	0.592	

Each model was adjusted for age, sex, educational level, marital status, prior cosmetic treatment history, and intended procedure category.

### 3.5 Mediation analysis: appearance stress as an indirect pathway

In the mediator model, maladaptive appearance perfectionism was strongly associated with higher appearance stress after covariate adjustment (*B* = 0.511, SE = 0.036, *p <* 0.001), whereas adaptive appearance perfectionism was not significantly associated with appearance stress (*B* = −0.018, SE = 0.040, *p =* 0.665). The mediator model explained 46.2% of the variance in appearance stress.

Bootstrap mediation analysis indicated statistically significant indirect effects of maladaptive appearance perfectionism on all four psychological outcomes through appearance stress. For appearance self-esteem, the indirect effect was −0.255 (95% bootstrap CI: −0.323 to −0.185), accounting for 52.7% of the total effect. For appearance satisfaction, the indirect effect was −0.234 (95% bootstrap CI: −0.301 to −0.178), accounting for 69.3% of the total effect. For social appearance anxiety, the indirect effect was 0.249 (95% bootstrap CI: 0.203 to 0.331), accounting for 54.1% of the total effect. For body image concern, the indirect effect was 0.243 (95% bootstrap CI: 0.176 to 0.328), accounting for 52.0% of the total effect. In all four models, the direct effect of maladaptive appearance perfectionism remained statistically significant after inclusion of appearance stress, supporting partial rather than complete mediation. The full model estimates presented in Table 5.

**Table 5:** Mediation models testing appearance stress as an indirect pathway between maladaptive appearance perfectionism and psychological outcomes.

Outcome	Total effect, c	Direct effect, c′	Stress to outcome path, b	Indirect effect, ab	95% bootstrap CI for ab	Proportion mediated
Appearance self-esteem	−0.483	−0.228	−0.498	−0.255	−0.323 to −0.185	0.527
Appearance satisfaction	−0.338	−0.104	−0.457	−0.234	−0.301 to −0.178	0.693
Social appearance anxiety	0.46	0.211	0.487	0.249	0.203 to 0.331	0.541
Body image concern	0.468	0.225	0.476	0.243	0.176 to 0.328	0.52

Common a path from maladaptive appearance perfectionism to appearance stress: *B* = 0.511, *SE* = 0.036, *p* < 0.001. The corresponding path from adaptive appearance perfectionism to appearance stress was not statistically significant (*B* = −0.018, *SE* = 0.040, *p* = 0.665). Indirect effects were estimated by bias-corrected bootstrapping.

## 4 Discussion

This single-center cross-sectional study investigated the distinct associations of maladaptive and adaptive appearance perfectionism with body image and psychosocial outcomes among adult patients seeking cosmetic dermatological treatments. The principal findings elucidate the dualistic nature of appearance-focused perfectionism in this clinical context. Maladaptive appearance perfectionism emerged as a robust and consistent risk factor, demonstrating significant negative associations with appearance self-esteem and satisfaction, while concurrently predicting heightened social appearance anxiety and body image concern. In stark contrast, adaptive appearance perfectionism was linked to more favorable body image constructs, specifically higher appearance self-esteem and satisfaction, without contributing to elevated anxiety or concern. A central and novel contribution of this investigation is the identification of appearance-related stress as a critical indirect psychological pathway. Maladaptive perfectionism was strongly correlated with elevated appearance stress, which in turn significantly mediated its detrimental effects on all four primary outcome variables. The results underscore that the pathway from rigid, self-critical perfectionism to body image disturbance in cosmetic patients is not direct but operates substantially through the experience of chronic, perfectionism-induced stress related to one’s looks.

The pattern of associations observed for maladaptive appearance perfectionism aligns closely with and extends a growing body of literature conceptualizing this construct as a core vulnerability factor for body image psychopathology. The strong, negative relationships with appearance self-esteem and satisfaction corroborate meta-analytic evidence demonstrating a clear link between BDD symptom severity and low global self-esteem, a connection that persists even after accounting for depressive symptoms (9). Our findings suggest that for individuals with maladaptive perfectionistic tendencies, negative self-evaluation appears generalized, impacting both global self-worth and domain-specific appearance esteem. The significant positive associations with social appearance anxiety and body image concern are also consistent with prior work. For instance, fear of negative appearance evaluation has been identified as a predictor of cosmetic surgery consideration (32), and our data indicate that maladaptive perfectionism may fuel this specific social anxiety. Furthermore, the correlation with elevated body image concern resonates with research identifying perfectionism as a salient feature in individuals with high dysmorphic concerns (19) and in those seeking aesthetic procedures (33). The strength of the associations in our model underscores the potency of this trait, potentially exceeding the influence of other known risk factors like gender or BMI, which have shown more variable relationships in previous studies (7, 18).

Concerning adaptive appearance perfectionism, our results provide empirical support for its conceptualization as a separate dimension with distinct, and potentially protective, correlates. Its positive associations with appearance self-esteem and satisfaction, in the absence of links to anxiety or excessive concern, offer a nuanced perspective. This pattern suggests that holding high personal standards for one’s appearance, when decoupled from critical self-evaluation and fear of imperfection, may be associated with a sense of competence and satisfaction in managing one’s looks. This differentiation is crucial, as it moves beyond a monolithic view of perfectionism as inherently pathological. Prior validation work on instruments like the Physical Appearance Perfectionism Scale (PAPS) has identified a “Worry about Imperfection” subscale that is more closely tied to pathology, while other facets may be less detrimental (17). Our findings are congruent with this view, indicating that the drive for an aesthetic ideal is not universally harmful; its impact depends on whether it is framed by self-criticism and perceived pressure or by personal agency and strivings. Different from maladaptive perfectionism, the adaptive dimension does not appear to inoculate against broader societal pressures, which are captured by other constructs like thin-ideal internalization (34, 35).

A pivotal contribution of this study is the empirical demonstration of appearance stress as a significant indirect pathway. The high correlation between maladaptive perfectionism and appearance stress, coupled with the substantial indirect effects through this mediator, provides a plausible explanatory model. Individuals with maladaptive appearance perfectionism likely experience a chronic state of stress and pressure centered on their appearance, driven by a perceived discrepancy between their actual and idealized self-image, fear of negative evaluation, and ruminative preoccupation (21). This sustained stress state then erodes self-esteem and satisfaction while amplifying anxiety and concern. This mediation pathway aligns with general psychological models where perfectionism predicts psychological distress via increased stress reactivity (36, 37). It also offers a specific instantiation for the cosmetic dermatology context, explaining how perfectionistic cognitions are associated with affective body image disturbances. The finding that adaptive perfectionism was not associated with this stress pathway further validates the distinction between the two dimensions; adaptive strivings do not trigger the same debilitating cycle of pressure and distress.

It should be noted that maladaptive appearance perfectionism and appearance stress were strongly correlated (*r* = 0.638), accounting for approximately 41% of shared variance. Although this suggests substantial conceptual overlap, the two constructs are theoretically and empirically distinguishable: perfectionism reflects a dispositional tendency toward exacting standards and self-criticism, whereas appearance stress captures the affective burden of appearance-related evaluation. This distinction is further supported by the differential pattern of associations observed for adaptive perfectionism, which was unrelated to appearance stress but showed unique positive associations with appearance self-esteem and satisfaction.

This explanatory framework is consistent with work by Do Bú et al. (52), who proposed a similar model linking neuroticism to anxiety and depression through stress and ruminative processes in patients with vitiligo. Their findings in a dermatological population support the broader premise that personality-level vulnerabilities may be associated with psychological distress through stress-related pathways, reinforcing the relevance of the indirect-effect approach adopted in the present study (52).

The psychological and behavioral mechanisms linking maladaptive perfectionism, appearance stress, and negative outcomes are likely multifaceted. Cognitively, the individuals may engage in chronic upward social comparison, particularly on social media platforms featuring idealized imagery (21, 38). This comparison process is exacerbated by a tendency to ruminate and catastrophize about perceived flaws (21). The resulting stress may manifest physiologically and emotionally, lowering threshold for distress when confronted with appearance-related stimuli. Behaviorally, this cycle can fuel compulsive checking, reassurance-seeking, and the pursuit of repeated or unnecessary cosmetic procedures in a futile attempt to alleviate the distress, a pattern often seen in BDD (26, 30, 39). The concept of “not just right” experiences (NJREs) may be particularly relevant here; the perfectionistic need for one’s appearance to feel “just right” can drive repetitive behaviors and intensify distress when this feeling is unattainable (40). Furthermore, interpersonal difficulties and poor emotion regulation, which are linked to body image dissatisfaction in aesthetic surgery seekers (41), may both stem from and exacerbate the stress generated by maladaptive perfectionism, creating a vicious cycle.

### 4.1 Implications

The findings carry important implications for clinical practice and public health initiatives within cosmetic dermatology. First, they underscore the necessity of moving beyond simple demographic risk assessments to include psychological screening for traits like maladaptive appearance perfectionism. Preoperative psychological screening is increasingly recommended to identify patients at risk for poor outcomes, including those with BDD or unmanaged mental health concerns (29, 42). Our data suggest that screening tools, whether comprehensive like the Cosmetic Readiness Questionnaire (CRQ) which includes a perfectionism subscale (30, 43), or focused, should capture this dimension. Identifying patients high in maladaptive perfectionism allows clinicians to initiate informed conversations about expectations, provide psychoeducation on the link between perfectionism and dissatisfaction, and refer for psychological support before proceeding with treatment. Second, the identification of appearance stress as a key pathway points to potential intervention targets. Cognitive-behavioral approaches that directly address perfectionistic thinking, reduce rumination, and build stress management skills could be highly beneficial. Brief self-compassion interventions have shown efficacy in reducing maladaptive perfectionism and improving body image (44, 45), and internet-based cognitive behavioral therapy for perfectionism (ICBT-P) has demonstrated effectiveness in reducing perfectionism components (46). Integrating such low-intensity psychological support into cosmetic dermatology clinics could improve patient preparedness and long-term satisfaction.

### 4.2 Strengths and limitations

A notable strength of this study is its focused investigation within a specific and clinically relevant population, adults actively seeking cosmetic dermatological interventions. This enhances the ecological validity of the findings compared to studies using general community or student samples. The simultaneous examination of both maladaptive and adaptive dimensions of appearance perfectionism provides a more nuanced understanding than studies treating perfectionism as a unitary construct. The application of bootstrapped mediation analysis to formally test the indirect role of appearance stress represents a methodological advancement, moving from correlation to proposed causation within the constraints of cross-sectional data. The use of multiple validated body image constructs allows for a comprehensive mapping of the perfectionism landscape.

Several limitations must be acknowledged when interpreting the results. The single-center, cross-sectional design inherently limits generalizability and precludes causal inference. The findings from one center may not be representative of cosmetic dermatology patients in other geographic or cultural settings. The temporal sequence implied by the mediation model, while theoretically plausible, cannot be confirmed; longitudinal or experimental designs are required to establish whether changes in perfectionism lead to changes in appearance stress and subsequent body image outcomes. Reliance on self-report measures introduces the potential for common method bias and social desirability effects. Although we studied adaptive perfectionism, its measurement and conceptual boundaries require further refinement; what is adaptive in one context may become maladaptive under stress. Residual confounding by unmeasured variables, such as underlying depressive or anxiety disorders, history of childhood trauma (37), or specific social media usage patterns (35, 47), could influence the observed relationships. Furthermore, as all variables were assessed via self-report at a single time point, common method variance may inflate the observed associations, and future research could benefit from incorporating multi-method or multi-informant approaches to address this limitation. The predominantly female composition of the sample (80.2%), while expected in this clinical population, limits generalizability to male cosmetic dermatology patients; future studies should examine whether gender moderates the observed associations between appearance perfectionism, stress, and psychosocial outcomes. Finally, while the sample size was adequate for the analyses conducted, it may limit the detection of smaller interaction effects or subgroup differences.

### 4.3 Future directions

Future research should prioritize longitudinal studies tracking cosmetic dermatology patients from consultation through post-treatment follow-up. This would clarify whether pre-existing maladaptive appearance perfectionism and high appearance stress predict poorer satisfaction with cosmetic outcomes, higher rates of revision procedures, or the onset or worsening of BDD symptoms (26). Experimental studies are needed to test the efficacy of targeted interventions, such as brief cognitive restructuring modules aimed at reducing perfectionistic thinking or stress-management techniques, delivered within the dermatology setting (48). Investigating the role of modern digital influences is also critical. Research should explore how engagement with photo-editing apps (49), exposure to specific beauty ideals on social media (34, 38), and even the “Zoom effect” from increased videoconferencing (50) interact with perfectionistic traits to amplify appearance stress. Cross-cultural comparisons would be valuable to understand how societal beauty standards and pressures modulate the psychological pathways. Finally, there is a need to develop and validate brief, clinically feasible screening tools specifically for appearance perfectionism and appearance stress to facilitate their integration into routine cosmetic practice, complementing existing BDD screens (14, 31, 51).

## 5 Conclusion

Among adult patients seeking elective cosmetic dermatology care, maladaptive appearance perfectionism is consistently associated with lower appearance self-esteem, lower appearance satisfaction, greater social appearance anxiety, and more severe body image concern, whereas adaptive appearance perfectionism shows a more limited and generally favorable pattern of association. Appearance stress also emerges as a significant indirect pathway linking maladaptive appearance perfectionism with all major psychological outcomes examined. Taken together, the findings suggest that the psychological burden carried by cosmetic dermatology patients is shaped not only by appearance concerns themselves but also by the perfectionistic style through which appearance is evaluated. From a clinical and public health perspective, brief psychological screening that captures maladaptive appearance perfectionism and appearance-related stress may help identify patients at elevated risk of distress, unrealistic expectations, and poor psychosocial adaptation. Integrating such screening into cosmetic dermatology practice may support more informed consultations, more appropriate referrals, and more patient-centered care. Future longitudinal and multicenter studies are needed to confirm temporal relationships, assess generalizability, and determine whether targeted psychological intervention can improve outcomes in this growing patient population.

## Data Availability

The raw data supporting the conclusions of this article will be made available by the authors, without undue reservation.
